# Proteomics study and protein biomarkers of malignant ventricular arrhythmia in acute myocardial infarction patients

**DOI:** 10.1002/ctm2.1435

**Published:** 2023-11-14

**Authors:** Jian‐Liang Zhang, Hai‐Tao Hou, Yu Song, Mu Guo, Xiao‐Cheng Liu, Qin Yang, Guo‐Wei He

**Affiliations:** ^1^ The Institute of Cardiovascular Diseases and Department of Cardiovascular surgery, TEDA International Cardiovascular Hospital Chinese Academy of Medical Sciences and Graduate School of Peking Union Medical College and Tianjin University Tianjin China; ^2^ Critical Care Unit, The Institute of Cardiovascular Diseases TEDA International Cardiovascular Hospital, Tianjin University Tianjin China

Dear Editor,

By constructing proteomics profile to identify the protein characteristics this study found that decreased TGFBI and increased vWF are the characteristics of proteins in the mechanism of development of malignant ventricular arrhythmias (MVA) combined with acute myocardial infarction (AMI). These innovative findings provide valuable insight into the diagnosis and pathogenesis in the development of MVA.

As acute syndrome of coronary artery disease, AMI has high morbidity and mortality. Ventricular arrhythmias (ventricular tachycardia and ventricular fibrillation) are the most common cause of death in AMI.^1^ Malignant VA (MVA) is frequent in both ST elevated^1^ and non‐ST elevated^2^ AMI patients and is associated with increased 30‐day mortality.^2^ The present study was based on our previous experience in proteomics^3^ to identify the differential plasma proteins in AMI patients with or without MVA using data independent acquisition (DIA) proteomics method (Figure 1).

**FIGURE 1:**
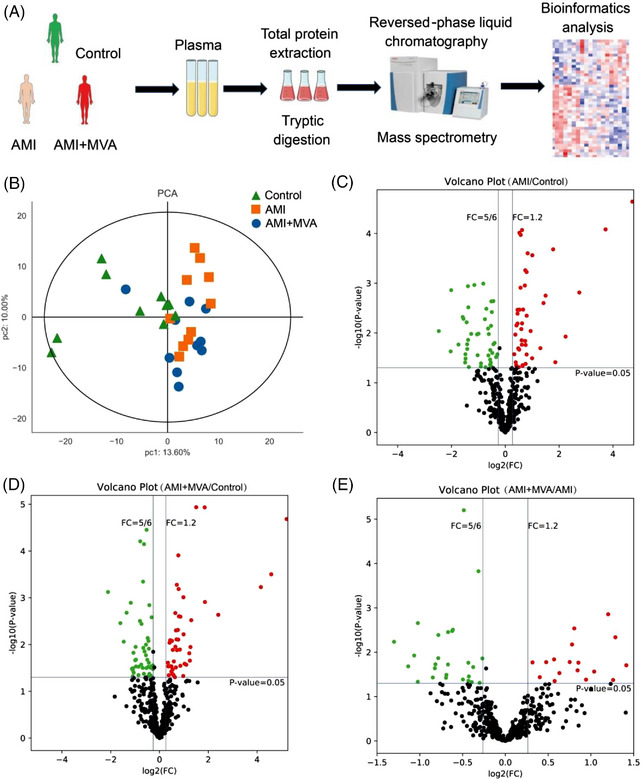
Overview of proteomics using data independent acquisition (DIA) method. (A) The schematic overview of the experimental approach. (B) Principal component analysis reveals a relative distinction of all the samples. (C–E) Volcano plot representing the profile of all the quantified proteins red, blue and black dots represent up‐regulation, down‐regulation and no significant change, respectively.

There were 3 groups (Control, AMI, and AMI +MVA). A total of 190 blood samples were allocated into these groups, and each had two phases: 30 samples for the proteomics discovery phase with 10 samples in each group and 160 samples for the validation phase (52 in Control, 54 in AMI and 54 in AMI +MVA group). Deferential proteins were revealed among three groups by one‐way ANOVA followed by Bonferroni post hoc test to detect the differences between two groups (AMI/control, AMI+MVA/control, and AMI+MVA/AMI).

The diagnostics criteria of AMI were defined as previously reported.^4^, ^5^

The characteristics of the patients are presented in Table S1.

Figure S1 is the flow chart of the experimental protocol. Figure 1A shows the overview of proteomics using DIA method. The principal component analysis (Figure 1B) reveals a relative distinction among the samples of 3 groups. A total of 8365 peptides and 460 proteins from the liquid chromatography‐tandem mass spectrometry analysis were identified. Figure 1C–E shows the profile of all the quantified proteins.

Of the identified proteins, 19.6% (90 of 460, Figure 2A) in AMI/control, 20.4% (94 of 460, Figure 2D) in AMI+MVA/control and 9.3% (43 of 460, Figure 2G) in AMI+MVA/AMI were screened as differentially expressed proteins (DEPs). Using DEPs, heatmap (Figure 2C,F,I) shows the relative abundance in each comparison group. Kyoto Encyclopedia of Genes and Genomes (KEGG) network diagrams are shown in Figure 2B, E, and H.

**FIGURE 2:**
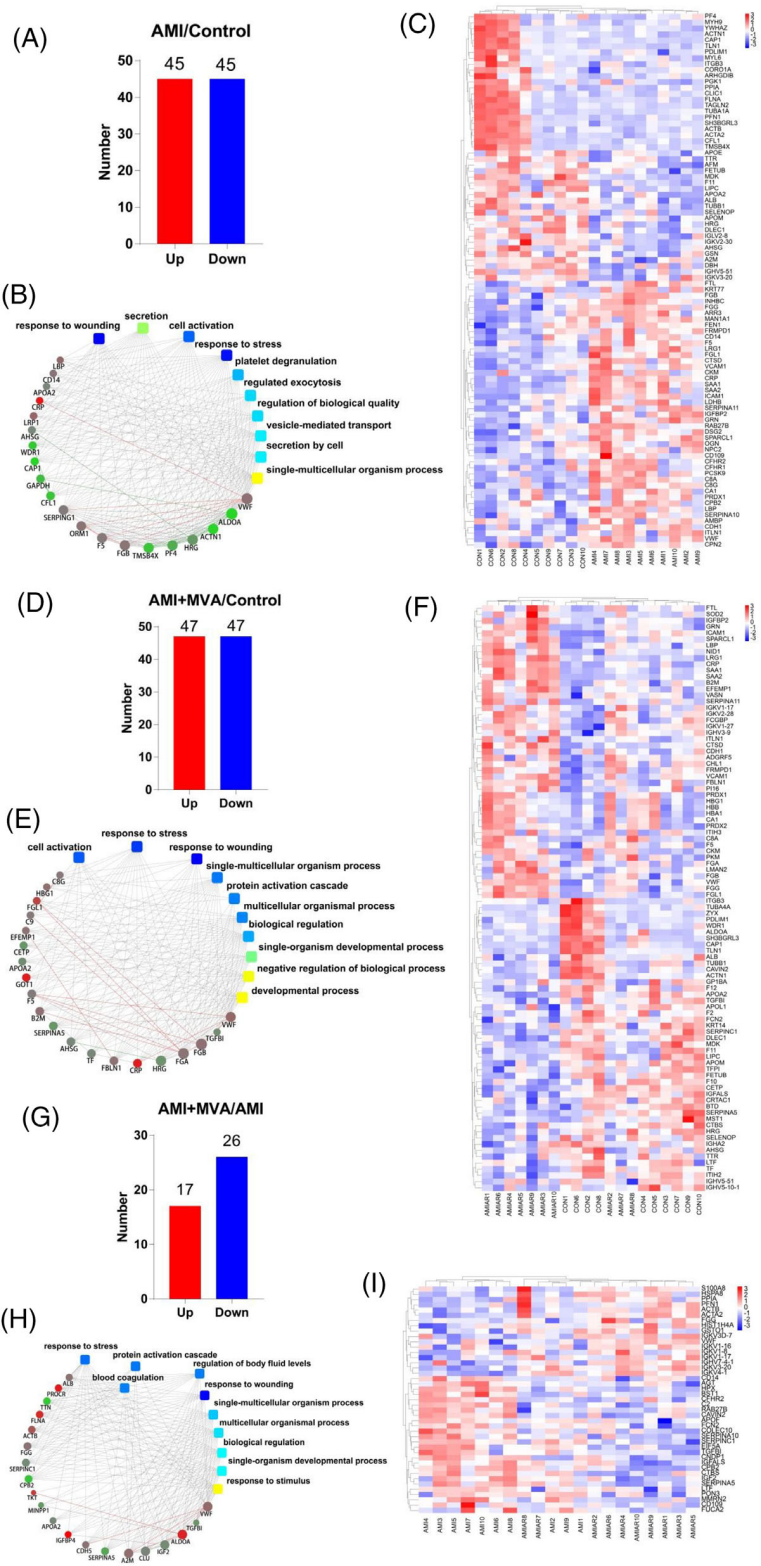
Analyses of differentially expressed proteins (DEPs). (A, D and G) Up‐regulated and down‐regulated proteins (*p* < .05, fold change > 1.2) in acute myocardial infarction (AMI)/control, AMI+malignant ventricular arrhythmias (MVA)/control, and AMI+MVA/AMI. (B, E and H) KEGG network diagrams show that DEPs are involved in 10 most significant corresponding KEGG pathways. (C, F and I) Heatmap displaying the relative abundance in each comparison using all the DEPs. The peptide of pooled samples was fractionated by 1100 HPLC System (Agilent). Q‐Exactive HF mass spectrometer with a Nanospray Flex source (Thermo Fisher Scientific) was used to analyze mass spectrometry (MS) data.

Venn diagram shows (Figure 3A) the overlap of DEPs based on three comparisons. There are significant differences regarding 2 proteins (vWF and FGG) in all three comparisons. A total of nine proteins (IGKV1‐17, TGFBI, FCN2, CTBS, SERPINA5, IGFALS, LTF, CAVIN2, and SERPINC1) show significant differences in AMI+MVA/control and AMI+MVA/AMI, but not in AMI/control. Heatmap of relative abundances of these 11 DEPs are shown in Figure 3B.

**FIGURE 3:**
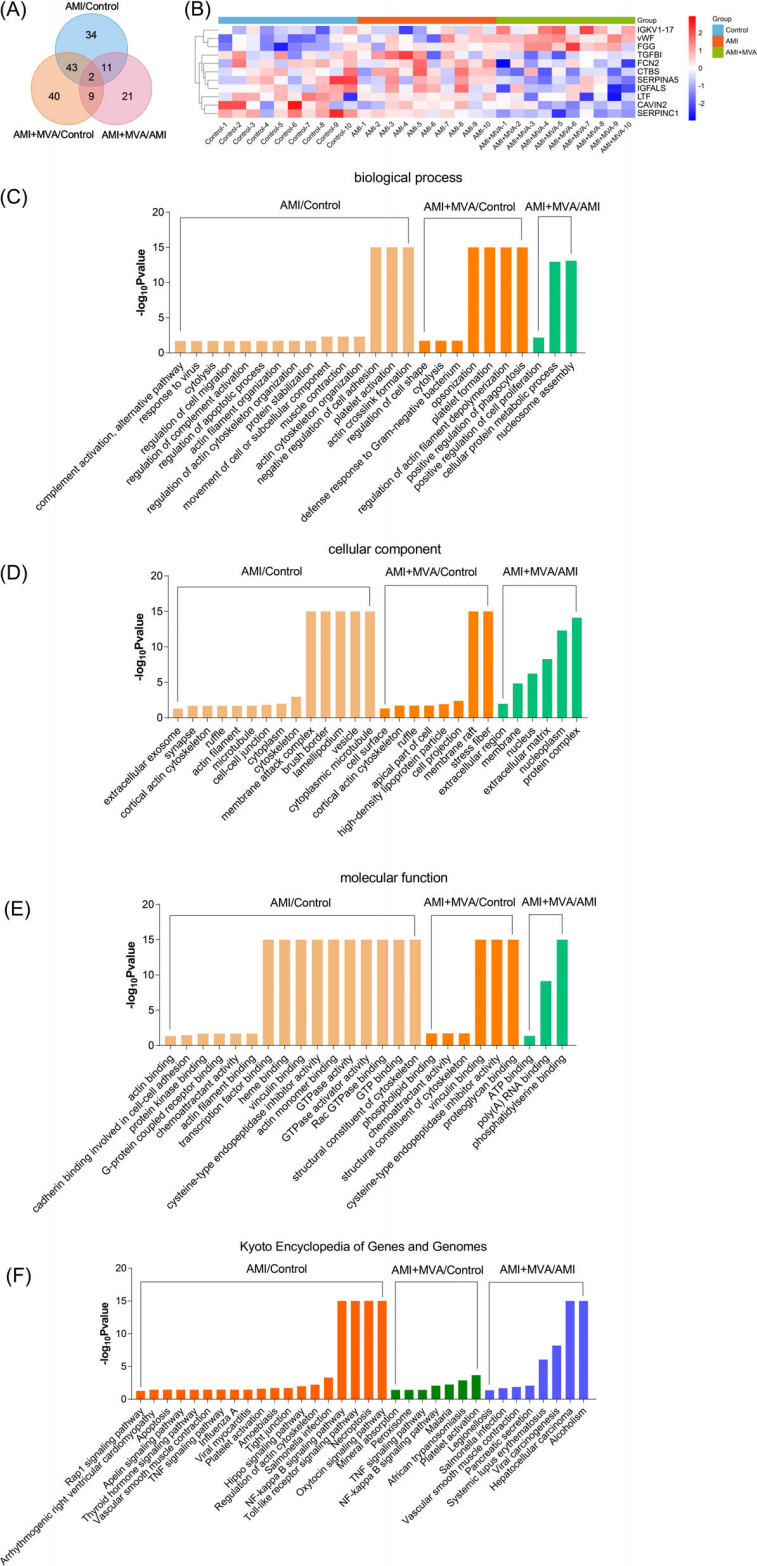
Bioinformatics analyses among acute myocardial infarction (AMI)/control, AMI+malignant ventricular arrhythmias (MVA)/control, and AMI+MVA/AMI. (A) Venn diagram showing the overlap of differentially expressed proteins (DEPs) on the basis of three comparisons. There are significant differences in two proteins (vWF and FGG) in all three comparisons. A total of nine proteins (IGKV1‐17, TGFBI, FCN2, CTBS, SERPINA5, IGFALS, LTF, CAVIN2, and SERPINC1) present significant differences in both AMI+MVA/control and AMI+MVA/AMI, but not in AMI/control. (B) Heatmap showing the relative abundance of above 11 DEPs. (C–F) The significantly different gene oncology (GO) categories (biological process, cell component, and molecular function) and KEGG pathways among three comparisons.

Gene oncology (GO) analyses including biological process, cellular component and molecular functions were also performed using DEPs. Significant GO terms (*p* < .05 and including at least 2 DEPs) were compared among three comparisons (Figure 3C–E).

The comparisons on KEGG were also performed with all significant pathways (Figure 3F), showing that legionellosis, pancreatic secretion, systemic lupus erythematosus, viral carcinogenesis, hepatocellular carcinoma and alcoholism were only found between AMI+MVA and AMI.

Figure 4A–C shows the intensity of TGFBI, vWF and ACTB, selected from the proteomics discovery phase by DIA technology (one‐way ANOVA and Bonferroni post hoc test). Further validations of these three proteins were performed in new cohort of patients by ELISA (Figure 4D–F, one‐way ANOVA and Bonferroni post hoc test). The ELISA validation results showed that TGFBI level significantly decreased in AMI+MVA (161.9 ± 19.0 ng/mL) compared to AMI (504.9 ± 39.9 ng/mL) or control (511.7 ± 49.1 ng/mL) (*p* < .0001, respectively) (Figure 4D).

**FIGURE 4:**
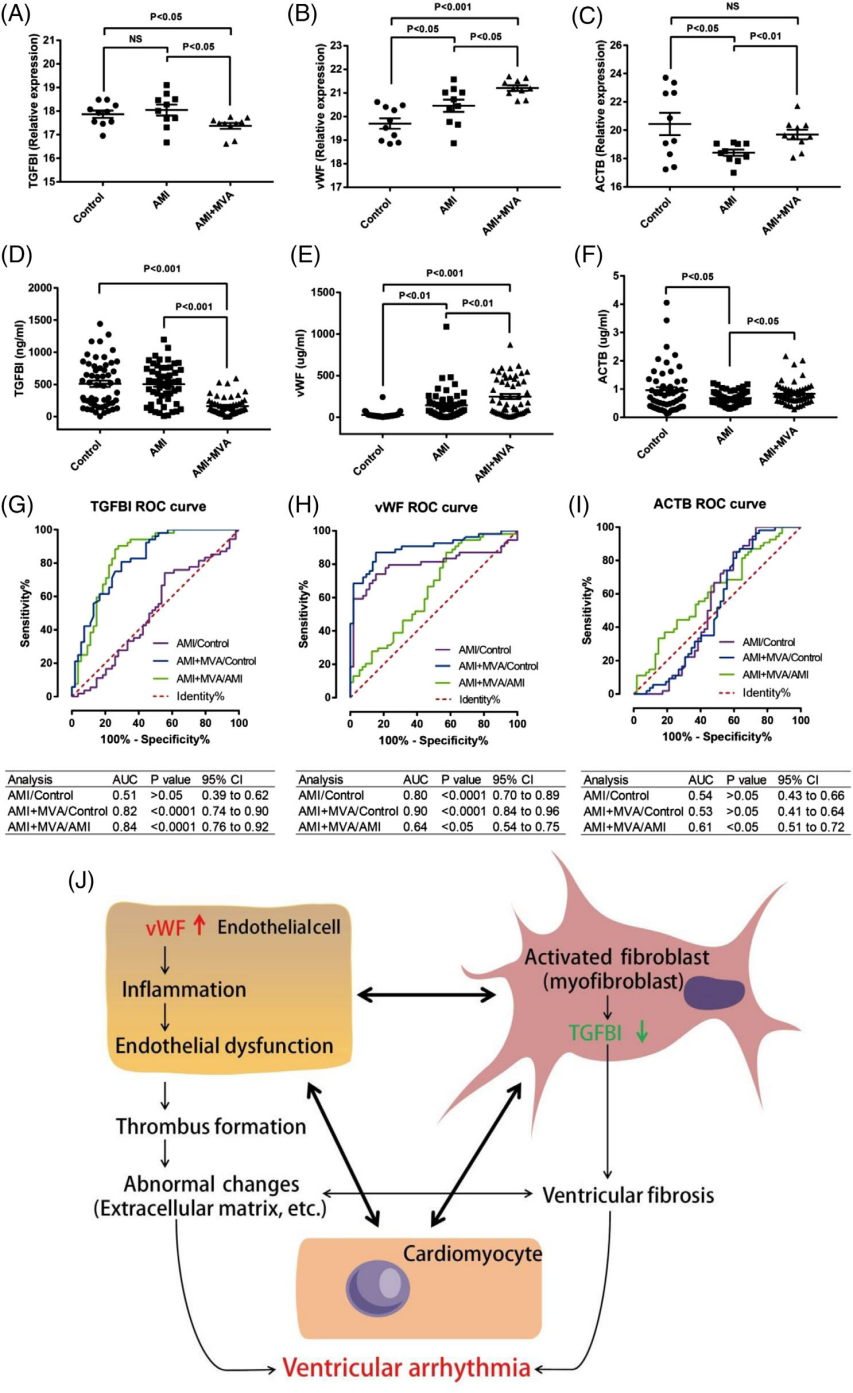
Validation of three differentially expressed protein candidates and schematic diagram to illustrate the role of TGFBI and vWF in the mechanism of development of ventricular arrhythmia. (A–C) Discovery phase by data independent acquisition technology. (D–F) Validation by enzyme linked immunosorbent assay in a new cohort of patients. TGFBI has the trend to fall from control to acute myocardial infarction (AMI)+malignant ventricular arrhythmias (MVA) while vWF has the trend to rise from control to AMI+MVA. (G–I) Receiver operating characteristic (ROC) analyses show that TGFBI and vWF have strong and potential value of predicting MVA in AMI. (J) Increased vWF results in various abnormal changes. These changes have interplay with ventricular fibrosis, which may be related to decreased TGFBI. Pathological changes caused by vWF and TGFBI dynamically contribute to the progression of ventricular arrhythmia.

Interestingly, vWF level has notable opposite trend in AMI+MVA patients (249.4 ± 29.9 µg/ml) compared with AMI patients (145.5 ± 24.1 µg/mL) and controls (28.8 ± 4.9 µg/mL) (Figure 4E; *p* < .01 or *p* < .0001, respectively. In addition, the ACTB level (µg/ml) in AMI patients (.69 ± .03) was markedly lower compared to controls (.96 ± .11; *p* < .05), but the level in the AMI+MVA patients (.84 ± .05 µg/mL) increased compared with AMI (.69 ± .03 µg/ml; *p* < .05) (Figure 4F).

In general, receiver operating characteristic (ROC) analysis shows that TGFBI, vWF and ACTB all have potential value of predicting MVA in AMI patients (Figure 4 G–I).

It was reported that over‐expression of TGFBI appears to support a greater cardiac growth response without impacting fibrosis.^6^ Interestingly, periostin was suggested as a marker of activated ventricular fibroblasts, in particular during development and in response to injury.^7^ Periostin and TGFBI are reported to have opposite roles^6^; therefore, it is possible that loss of TGFBI may contribute to fibrosis (Figure 4J). In this study, TGFBI in AMI+MVA significantly decreased compared with control or AMI group. We infer that decreased TGFBI may be involved in the process of fibrosis and result in initiation and occurrence of arrhythmias. Taken together, TGFBI may be a novel biomarker of development of MVA in AMI patients.

vWF is closely correlated to another arrhythmia—atrial fibrillation (AF) that has higher plasma vWF compared to sinus rhythm.^8^ Assessment of plasma vWF also indicates a correlation between AF and endothelial dysfunction.^8^ Increased endothelium‐associated vWF is a major factor and represents a therapeutic target in early atherogenesis.^9^ Further, it was reported that overexpression of vWF in endocardium may occur in atrial structural remodeling, related to AF and thrombosis.^10^

Nevertheless, no study has reported the role of vWF to MVA in AMI patients. In this study, we found that significant higher concentration of vWF in AMI+MVA compared to AMI group that revealed the potential importance of vWF in the development of MVA (Figure 4J). This study for the first time reveals that vWF changes in MVA and is a potential biomarker for early diagnosis of MVA.

In conclusion, we found that decreased TGFBI and increased vWF are the characteristics of proteins in the MVA patients with AMI and are potential predicting biomarkers. Further study should be carried out to investigate the practical use of these biomarkers. Our findings provide novel perception in the understanding of mechanism of MVA in AMI and have clear clinical significance in diagnosis and treatment of MVA in AMI.

## CONFLICT OF INTEREST STATEMENT

The authors have nothing to disclose with regard to commercial support.

## FUNDING INFORMATION

The National Natural Science Foundation of China Grant Number: 82170353 and 82370350; Tianjin Science and Technology Commission, Grant Number: 22ZYQYSY00020 and 21JCYBJC01120; The Non‐profit Central Research Institute Fund of Chinese Academy of Medical Sciences, Grant Number: 2020‐PT310‐007; Tianjin Key Medical Discipline (Specialty) Construction Project, Grant Number: TJYXZDXK‐019A

## Supporting information

Supporting Information

## Data Availability

The mass spectrometry proteomics data have been deposited to the ProteomeXchange Consortium via the PRIDE partner repository with the dataset identifier PXD041535
