# Supplementary material for: Proteomics study and protein biomarkers of malignant ventricular arrhythmia in acute myocardial infarction patients
Source: Clin Transl Med. 2023 Nov 14;13(11):e1435. doi: 10.1002/ctm2.1435 (PMC10644326; doi:10.1002/ctm2.1435)
Supplement: Supplementary file 1 — Supporting Information [file CTM2-13-e1435-s001.docx]

September, 2023

Letter to the Journal # CTM2-2023-06-1071

for submission to ***Clinical and Translational Medicine***

**Proteomics Study and Protein Biomarkers of Malignant Ventricular Arrhythmia in Acute Myocardial Infarction Patients**

**Running title:** **Biomarkers of Ventricular Arrhythmia in AMI**

Jian-Liang Zhang,^a^ Hai-Tao Hou,^a^ Yu Song,^a^ Mu Guo,^a^ Xiao-Cheng Liu^a^, Qin Yang,^a^ and Guo-Wei He^a^*

^a^The Institute of Cardiovascualr Diseases &Department of Cardiovascular surgery, TEDA International Cardiovascular Hospital, Chinese Academy of Medical Sciences & Graduate School of Peking Union Medical College &Tianjin University, Tianjin, China

^*^Correspondence author:

**Professor Guo-Wei He, *MD, PhD, DSc***

Distinguished Professor of Tianjin University, China

Foreign Correspondence Member, The National Academy of Medicine, France

Clinical Professor of Surgery, OHSU, Portland, OR, USA

No.61, 3rd Ave, TEDA Int’l CV Hosp., TEDA, Tianjin, 300457, CHINA

Tel: 86-22-65209089; Fax: 86-22-65208732

E-mail: [gwhezj@163.com](mailto:gwhezj@163.com) or gwhe@tju.edu.cn

**Figure S1.** Consolidated Standards of Reporting Trials (CONSORT) diagram of the study.

**Table S1.** Patient characteristics of the proteomics/validation cohorts among three groups.

**Figure S1.**


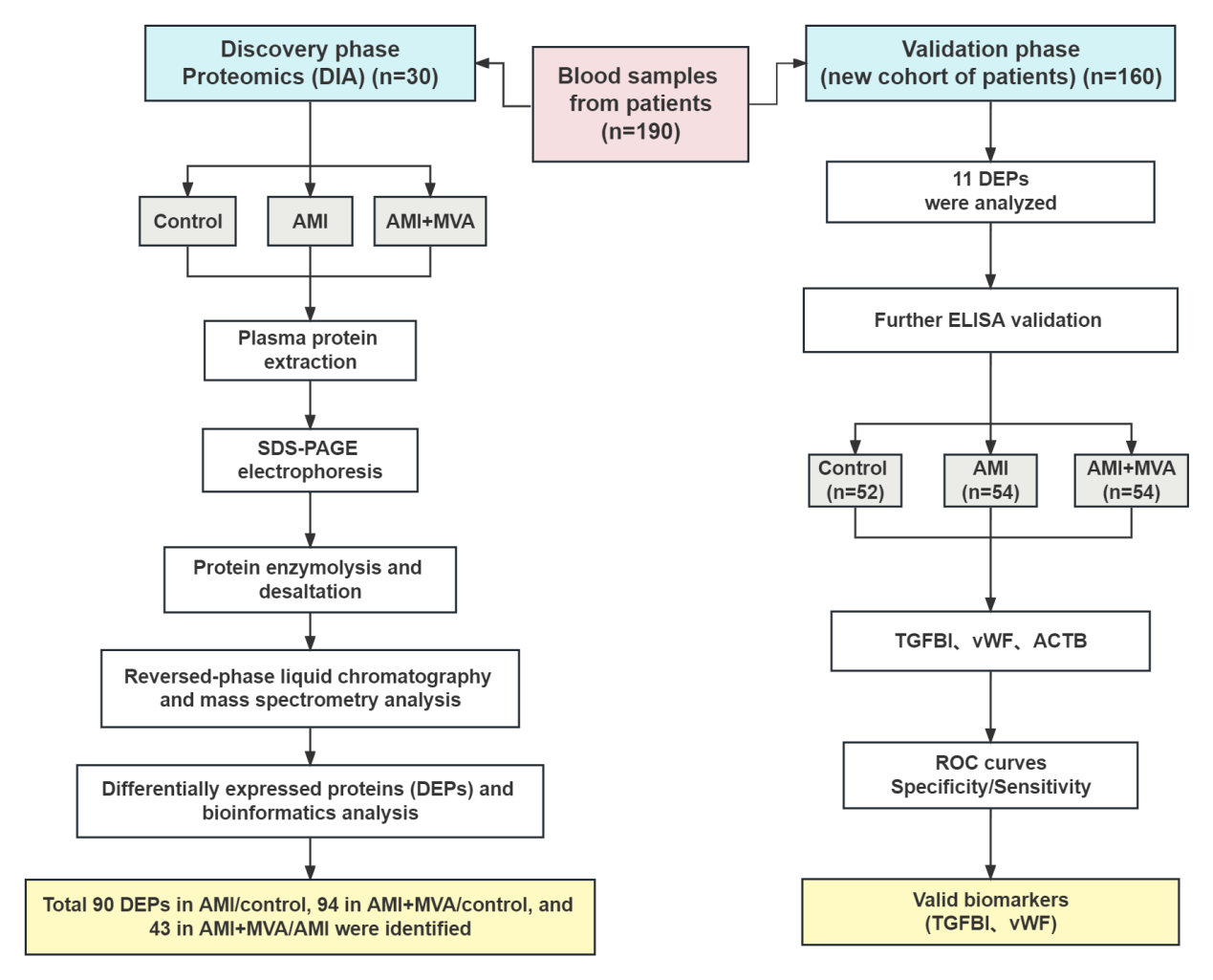


**Figure S1.** **Consolidated Standards of Reporting Trials (CONSORT) diagram of the study**. AMI, acute myocardial infarction; MVA, malignant ventricular arrhythmias; DIA, data independent acquisition, ROC, receiver operating characteristic.

**Table S1.** **Patient characteristics of the proteomics/validation cohorts among three groups.**

| Variables | Proteomics patients | | |  | Verification patients | | | | |  |
| --- | --- | --- | --- | --- | --- | --- | --- | --- | --- | --- |
|  | Control  (n=10) | AMI  (n=10) | AMI+MVA  (n=10) | *P* |  | Control  (n=52) | AMI  (n=54) | AMI+MVA  (n=54) | *P* | |
| Age | 62.3±5.1 | 69.4±12.7 | 72.2±9.7 | 0.08 |  | 60.1±7.5 | 62.8±11.3 | 66.8±12.2 | <0.01 | |
| BMI | 24.0±3.0 | 23.8±2.7 | 25.1±2.1 | 0.90 |  | 25.9±2.9 | 25.2±3.0 | 25.4±4.1 | 0.94 | |
| Smoking(%) | 5（50） | 5（50） | 7（70） | 0.58 |  | 17(34) | 25(47.2) | 31(57.4) | 0.04 | |
| Drinking(%) | 5（50） | 5（50） | 2（20） | 0.29 |  | 10(20) | 11(20.8) | 11(20.4) | 0.76 | |
| Hypertension(%) | 5（50） | 8（80） | 7（70） | 0.35 |  | 27(54) | 35(66.0) | 40(74.07) | 0.05 | |
| Hyperlipidemia(%) | 0（0） | 1（10） | 0（0） | 0.36 |  | 9(18) | 0(0) | 1(1.9) | <0.01 | |
| diabetes(%) | 3（30） | 4（40） | 0（0） | 0.90 |  | 10(20) | 16(30.2) | 10(18.5) | 0.09 | |
| LVDD(mm) | 46±3.9 | 45.8±1.9 | 50.6±4.5 | 0.01 |  | 46.0±2.7 | 47.8±4.5 | 47.61±5.5 | 0.09 | |
| LVPW(mm) | 9.4±0.5 | 9.7±0.5 | 9.7±0.7 | 0.41 |  | 9.7±0.7 | 9.7±0.6 | 9.9±0.5 | 0.05 | |
| LVEF (%) | 64.5±5.9 | 63.3±4.7 | 51.8±7.4 | <0.01 |  | 65.2±4.4 | 56.5±9.2 | 54.4±10.4 | <0.01 | |
| Cr(mmol/L) | 57.4±12.7 | 65.1±14.6 | 70.±33.1 | 0.41 |  | 61.1±13.7 | 72.6±56.3 | 82.35±31.8 | 0.02 | |
| K^+^(mmol/L) | 4.1±0.4 | 3.49±0.9 | 3.8±0.5 | 0.15 |  | 4.1±0.3 | 3.9±0.4 | 4.02±0.4 | 0.05 | |
| HGB(g/L) | 137.4±12.7 | 122.7±23.9 | 135.9±17.6 | 0.18 |  | 136.1±13.7 | 140.±22.2 | 126.4±16.8 | <0.01 | |

BMI, body massive index; LVDD, left ventricular diastolic disorder; LAPW, left atrial posterior wall; LVEF, left ventricular ejection fraction: HGB, hemoglobin
